# Antioxidation is a Common Defense Strategy for Different Species Under Mechanical Stress

**DOI:** 10.1002/ggn2.202500043

**Published:** 2026-03-13

**Authors:** Feixiang Zhu, Kai Huang

**Affiliations:** ^1^ Institute of Mechanobiology & Medical Engineering School of Life Sciences & Biotechnology Shanghai Jiao Tong University Minhang Shanghai China

**Keywords:** antioxidant strategy, mechanical stress, mechanotransduction, oxidative stress

## Abstract

Mechanical stress, a ubiquitous physical stimulus, triggers oxidative stress through mechanotransduction in diverse organisms, driving the evolution of antioxidant defense systems. This perspective deciphers the molecular evolutionary principles governing these antioxidant strategies, uncovering both conserved and lineage‐specific adaptations across biological kingdoms. We demonstrate that redox regulation—an ancient and evolutionarily conserved mechanism—serves as a core defense against mechanical stress‐induced oxidative damage, albeit with kingdom‐specific variations. Notably, mechanosensors such as Piezo channels are conserved in animals and plants, initiating antioxidant responses via Ca^2^^+^ signaling. While animals rely on the Nrf2 regulatory axis to upregulate antioxidant enzymes, plants deploy intricate networks involving SOD, CAT, and non‐enzymatic antioxidants (e.g., ascorbic acid). In contrast, extremophilic microorganisms employ streamlined yet highly efficient strategies, including multifunctional molecules like DMSP, to mitigate oxidative stress. Our comparative analysis highlights the evolutionary significance of these adaptations, shaped by ecological pressures and physiological constraints. Beyond elucidating life's evolutionary trajectory, these insights hold translational potential—from enhancing crop stress tolerance to informing biomaterial innovations and therapeutic interventions for oxidative disorders.

## Graphical abstract

Mechanical stress‐induced oxidative stress is a universal challenge across species, triggering a conserved antioxidant defense strategy. This perspective delves into the molecular evolutionary logic of this response, comparing mechanisms across plants, microorganisms, and animals to reveal their profound phylogenetic significance. Ultimately, these evolutionary insights provide a theoretical foundation for enhancing crop resilience, guiding biomaterial design, and developing therapies for oxidative pathologies.

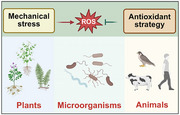

## 1 Introduction

In the course of Earth's evolution, the emergence of molecular oxygen served as a pivotal selective pressure driving the evolution of antioxidant defense systems. With the rise of aerobic respiration, organisms were exposed to higher levels of molecular oxygen, which prompted the gradual evolution of more efficient antioxidant defense mechanisms. These systems further developed and diversified during subsequent evolution in response to the demands of different ecological niches, stresses, and physiological functions. Within animal cells, reactive oxygen species (ROS)‐an umbrella term encompassing oxygen‐derived free radicals such as superoxide anion (O_2_^−^), hydrogen peroxide (H_2_O_2_), and hydroxyl radical (·OH)‐are generated through multiple enzymatic and metabolic pathways [1]. In addition to the well‐characterized NADPH oxidase (NOX) and mitochondrial electron transport chain (ETC), other enzymatic sources—such as peroxidases, nitric oxide synthase, lipoxygenases, xanthine oxidase, glucose oxidase, myeloperoxidases, and cyclooxygenases‐also contribute significantly to intracellular ROS production [1]. In plant cells, ROS signaling can be derived from normal metabolic processes in chloroplasts and mitochondria, peroxisomes, the endoplasmic reticulum, or plasma membrane‐localized respiratory burst oxidase homologs (RBOHs), which belong to the NOX family [2, 3]. Different ROS exhibit distinct chemical properties and biological functions. These diverse ROS‐producing pathways are activated in response to various stimuli, including mechanical stress, collectively modulating the cellular redox balance. In the broader evolutionary framework, mechanical stress—a pervasive physical stimulus—acts as both a catalyst and a sculptor of adaptation. While not the original driver behind the emergence of antioxidant systems, mechanical stress amplifies intracellular redox imbalances, imposing strong selective pressure on ancestral antioxidant defenses. Over time, this dynamic has critically shaped the adaptive strategies of diverse life forms throughout evolution. From the fluid shear stress and hydrostatic pressure acting on marine microorganisms [4, 5], to the mechanical resistance encountered by plant roots penetrating compacted soil [6], and the shear stress and cyclic stretching exerted on the cardiopulmonary vascular system by blood flow or external stress in animals [7, 8, 9], mechanical stress is a pervasive physical factor in the biological survival environment. While these forces are crucial for maintaining tissue structure, enabling movement, and facilitating ecological interactions, they also trigger profound biochemical responses that go beyond structural adaptations. Notably, oxidative stress induced by mechanotransduction has emerged as a conserved pathophysiological challenge [8, 10, 11], significantly regulating cellular viability and organismal adaptability.

At the subcellular level, mechanical stimuli can inflict oxidative damage through a variety of mechanisms. First, membrane perturbation can disrupt the normal functions of transmembrane proteins, including Piezo1 [12], NOX2 [8], and NINJ1 [13]. Second, it can mediate changes in organelle morphology, which in turn affect the efficiency of the mitochondrial ETC [7, 14] and the protein‐folding capacity of the endoplasmic reticulum (ER) [10, 15]. Third, cytoskeletal remodeling serves as a regulator for redox‐sensitive signaling pathways [16]. These perturbations can set off a chain reaction, ultimately leading to redox imbalance. Under physiological conditions, a moderate increase in ROS levels can actually be beneficial, promoting the metabolism, synthesis, and secretion of cytokines and other effector molecules. However, when ROS levels become excessively high, they transform into “cellular saboteurs”. These high ‐ level ROS attack biomacromolecules such as lipids, proteins, and nucleic acids, causing oxidative damage. This damage disrupts normal cellular physiological functions and, in severe cases, can lead to cell death. Ultimately, this may result in tissue dysfunction or even pathological changes [17, 18].

Despite these challenges, throughout the course of prolonged evolution, various species have developed unique antioxidant mechanisms. The evolutionary convergence of antioxidant mechanisms across different biological kingdoms strongly suggests that redox regulation is a fundamental and core strategy for coping with the oxidative challenges induced by mechanical stress, although the specific ways in which this is implemented vary among species. Even though different species have evolved diverse antioxidant systems in response to their specific ecological pressures, such as the presence of phenolic polymers in plants [19, 20] and heat shock proteins in animals [21], the core principles of neutralizing ROS through enzymatic (e.g., superoxide dismutase, catalase) and non‐enzymatic (e.g., ascorbic acid, glutathione) systems remain relatively conserved [19, 20, 21, 22, 23]. To ensure the robustness of our comparative analysis of antioxidant mechanisms across species, we conducted a semi‐systematic literature review. This perspective integrates cross‐species evidence to elucidate that antioxidant defense represents a shared evolutionary solution for addressing the biochemical consequences of mechanical stress. By dissecting the molecular interaction mechanisms between mechanotransduction and redox homeostasis in plants, microorganisms, and animals, we aim to clarify how biological systems remodel antioxidant networks to transform mechanical vulnerability into adaptive resilience.

## 2 Molecular Evolutionary Logic of Antioxidant Defense

Mechanical stress converts physical challenges into redox crisis at the cellular level via a multi‐tiered molecular network, with the crux being the conservatism of “mechanical‐chemical” signal transduction. When cells sense mechanical stimuli, the activation of NADPH oxidases (e.g., the NOX2 complex) triggers a burst of ROS [8]. Concurrently, cytoskeletal rearrangement disrupts the structure of mitochondrial cristae, leading to respiratory chain blockage [14]. This conserved ROS‐generating mechanism offers an ideal model for exploring the molecular evolution of antioxidant defense. By comparing the response strategies of different species to mechanical stress, we can elucidate how life strikes a balance between “primitive conservatism” and “environmental adaptability”.

During Earth's early history, the atmosphere was nearly devoid of oxygen, and life relied predominantly on anaerobic metabolism. However, the advent and expansion of photosynthetic organisms triggered a profound shift—oxygen levels gradually rose, exposing life to unprecedented oxidative stress. This transition forced organisms to confront the dual challenge of harnessing oxygen's metabolic advantages while mitigating its damaging effects [24, 25]. To adapt to this challenge, organisms evolved sophisticated antioxidant defense systems. Over time, these mechanisms underwent continuous refinement and diversification, shaped by varying ecological pressures and physiological demands across different species. For example, organisms in high‐oxygen environments often develop stronger antioxidant defenses to counteract oxidative stress, while those in low‐oxygen conditions may possess less robust antioxidant systems but still undergo adaptive modifications to meet their specific physiological demands [26, 27].

Redox homeostasis and signaling are pivotal in driving evolutionary adaptation across organisms. The intracellular redox state regulates both cellular physiology and signaling transduction pathways. Mechanical stress disrupts intracellular redox balance, inducing oxidative stress. This oxidative stress acts as a signaling cue, triggering antioxidant defense mechanisms and activating relevant signaling pathways to modulate cellular physiology and gene expression, thereby enhancing environmental adaptation. Exercise‐induced skeletal muscle adaptation is mechanistically linked to the type of mechanical stress encountered. In endurance exercise, upregulated aerobic metabolism escalates electron leakage from mitochondrial respiration, fostering superoxide overproduction and consequent oxidative stress—a key driver of adaptive signaling [28]. ROS act as pleiotropic regulators, activating both the Nrf2‐ARE pathway (to enhance antioxidant responses) and the PI3K/Akt/mTOR axis (to stimulate protein synthesis). The resultant antioxidant upregulation and myofiber hypertrophy synergistically improve muscle strength and endurance, exemplifying ROS‐dependent mechanotransduction in exercise adaptation [29, 30]. Evolutionary analysis unveils the following threefold molecular logic in the response to mechanical stress: primitive commonality, eukaryotic divergence, and animal specialization.

### 2.1 Primitive Commonality

A substantial number of eukaryotes share core components for mechanical force perception. Mechanically activated Piezo channels and other mechanosensitive ion channels (MSCs) act as conserved mechanical sensors. Their gene structures display homologous conservatism in both animals and plants, establishing the molecular basis for cross ‐ kingdom responses [6, 31, 32]. In particular, the Ca^2+^ influx mediated by Piezo1 is closely associated with oxidative stress responses [33]. For bacteria, they encounter contact forces, hydrostatic pressure, and shear forces in their natural habitats. Structures such as the cell envelope, flagella, and pili transmit these forces through their mechanical properties. Although it is still uncertain whether the “mechanical sensors” (such as Piezo channels) conserved in animals and plants are applicable to bacteria at present, bacteria have also developed a unique set of strategies to cope with mechanical stress over the course of long‐term evolution. From an evolutionary perspective, the antioxidant strategies of bacteria exhibit both differences and certain similarities to the “conserved core” of eukaryotic organisms. The antioxidant strategies in bacteria tend to follow a minimalist approach, having evolved based on their relatively simple cellular structure and characteristics of rapid reproduction and environmental adaptability. In summary, transforming mechanical stimuli into eliminable oxidative stress is a common strategy for organisms to cope with external forces. In summary, mechanotransduction‐mediated oxidative stress and downstream signaling constitute a fundamental adaptive mechanism by which organisms respond to external mechanical forces.

### 2.2 Eukaryotic Divergence

With the divergence of the plant and animal kingdoms, mechanical response mechanisms have undergone convergent evolution. In animals, FoxO/Nrf2 transcription factors [34, 35], and in plants, NAC family proteins [36] have all acquired the ability to perceive mechanical stimuli. Nevertheless, significant differences exist in their regulatory targets. Animals tend to upregulate glutathione synthetase [37, 38], while plants enhance the efficiency of the ascorbate‐glutathione cycle (AsA‐GSH) [39].

### 2.3 Animal Specialization

In the subphylum Vertebrata, the coupling between mechanical stress and the immune system has emerged as a prominent characteristic. For instance, under mechanical stimulation, macrophages experience an increase in cytoplasmic and mitochondrial ROS production. This impairs their efferocytosis function and induces sterile inflammation [40].

This molecular architecture, characterized by a “conserved core and variable periphery”, elucidates the dual nature of the animal antioxidant system in response to mechanical stress. It not only retains the core components inherited from primitive eukaryotes but also achieves adaptive compensation through mechanisms such as transcriptional regulation. The interaction between basic research and clinical practice further strengthens this duality. In ‐ depth analysis of regulatory mechanisms provides novel targets for medical interventions like antioxidant supplementation, ultimately forming a unique interactive model of evolutionary heritage and medical innovation.

## 3 Comparative Analysis of Antioxidant Mechanisms Across Species

**FIGURE 1:**
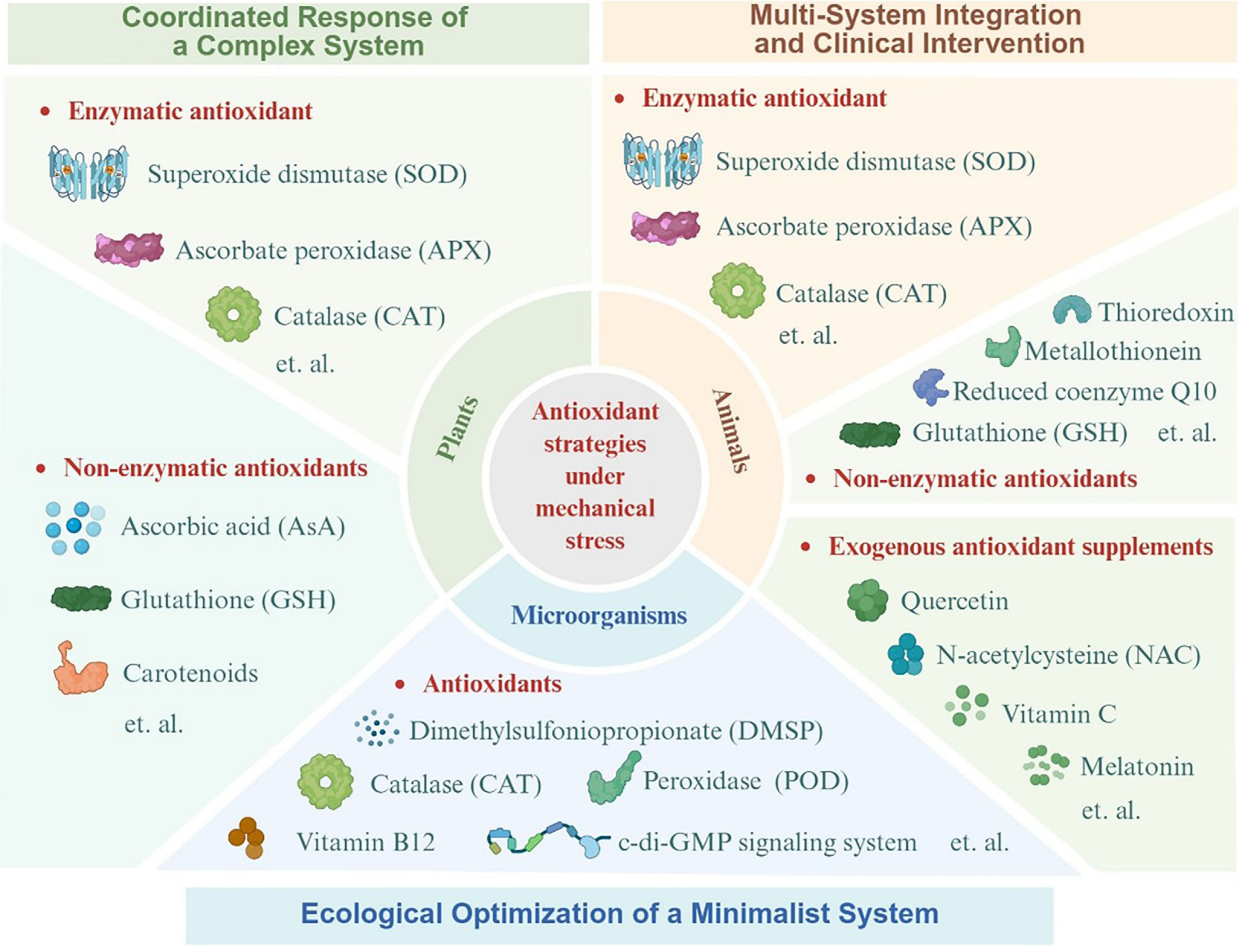
Antioxidant mechanisms across species in response to mechanical stress‐induced oxidative stress. Across the tree of life, organisms have evolved diverse antioxidant strategies to counteract mechanically induced oxidative stress—from the sophisticated redox‐regulatory networks of plants and the streamlined adaptations of extremophilic microorganisms, to the integrated, multi‐tiered defense systems of mammals, which are further augmented by clinical therapeutics in humans. These adaptations continually reshape how organisms perceive, respond to, and ultimately harness mechanical forces within their ecological niches.

The diversity of antioxidant strategies reflects the adaptive evolution of organisms in response to mechanical environments. By dissecting the defense systems of plants, microorganisms, and animals, as shown in Figure 1, we can uncover the modular architecture of redox regulation and its ecological plasticity.

### 3.1 Antioxidant Defense in Plants: Coordinated Response of a Complex System

During natural growth, plants inevitably encounter various types of mechanical stress, such as the pulling force exerted by strong winds, the impact force of heavy rain, and the external forces generated by animal grazing or touching. Meanwhile, plants also encounter common abiotic stresses such as drought, high temperature, cold, nutrient deficiency, as well as excessive salt or toxic metal content in the soil. ROS play a crucial role in stress perception, the integration of different stress‐response signaling networks, and the activation and acclimation of plant defense mechanisms [41]. Plants have evolved a sophisticated and efficient antioxidant defense system to cope with diverse stresses. From a correlative perspective, diverse stress conditions trigger ROS accumulation in plants, which in turn activates antioxidant defense systems. As central signaling molecules, ROS mediate stress perception, integrate signaling networks, and initiate defense responses. Regardless of stress type, plants must scavenge excess ROS via antioxidant mechanisms to preserve redox homeostasis and safeguard cellular integrity and function [42]. However, there are significant differences in the specific mechanisms between mechanical stress responses and general abiotic stress responses. Mechanical stress, such as external forces generated by strong winds or animal grazing, primarily acts directly on plant cell structures, causing physical disturbances in the cell membrane, rearrangement of the cytoskeleton, and alterations in organelle morphology, thereby affecting normal cellular physiological functions [43]. These physical changes rapidly trigger a series of unique signal transduction pathways, ultimately leading to the activation of antioxidant defense systems [44, 45]. In contrast, general abiotic stresses, such as drought and salt stress, primarily affect cellular metabolic processes and redox status indirectly by altering physiological parameters in plant cells, including water status, ion balance, and osmotic pressure [46]. Although these stresses also induce ROS accumulation, their triggering mechanisms and signal transduction pathways differ from those of mechanical stress. Plants have evolved a sophisticated and efficient antioxidant defense system to cope with various stresses, with numerous unique regulatory mechanisms specifically tailored to mechanical stress.

In plants, there exist specific signaling pathways dedicated to perceiving mechanical stimuli, and these pathways are uniquely activated under mechanical stress. For instance, mechanically activated Piezo channels and other MSCs, serving as conserved mechanosensors, mediate Ca^2+^ influx upon sensing mechanical stimuli, thereby triggering a series of downstream signaling events [47]. When plants suffer mechanical damage, the intracellular Ca^2+^ concentration rises rapidly, which subsequently activates the antioxidant enzyme system and enhances the plant's resistance to mechanical stress [48].

In the enzymatic antioxidant defense line, superoxide dismutase (SOD) takes the lead by rapidly converting superoxide anions into hydrogen peroxide and oxygen, thereby reducing the cytotoxicity of superoxide anions [49, 50, 51, 52]. Subsequently, catalase (CAT) and ascorbate peroxidase (APX), among others, take over. CAT decomposes hydrogen peroxide into water and oxygen, while APX, using ascorbic acid as an electron donor, catalyzes the reduction of hydrogen peroxide, further eliminating hydrogen peroxide to prevent more severe damage to cells [19, 51].

Compared with general abiotic stresses, mechanical stress can directly act on plant cell structures, inducing dynamic changes in ROS, which may result in a more direct and specific activation and regulation of the plant antioxidant defense system. Following mechanical damage to plants, the elevated ROS levels rapidly activate the activities of antioxidant enzymes such as SOD and CAT, while simultaneously promoting the synthesis of non‐enzymatic antioxidants like ascorbic acid (AsA) and glutathione (GSH). This collectively forms an efficient antioxidant defense network that safeguards plant cells from oxidative damage. Non‐enzymatic antioxidants are also essential components of the plant antioxidant defense system. AsA not only directly reacts with ROS to scavenge them but also serves as a substrate for enzymes like APX, supporting enzymatic reactions. GSH possesses potent antioxidant capacity, protecting biomacromolecules from oxidative damage, participating in intracellular signal transduction and metabolic regulation, and maintaining cellular redox balance. Carotenoids can quench singlet oxygen and scavenge free radicals, reducing oxidative stress‐induced cellular damage [19, 51].

In general, when plants are subjected to mechanical stress, their antioxidant defense system rapidly responds and works in concert. On one hand, it efficiently scavenges excessive ROS generated within cells, preventing damage to cellular structures and functions, protecting the integrity of cell membranes, maintaining the activity of various enzymes in cells, and ensuring normal physiological processes such as material synthesis and energy conversion. On the other hand, ROS generated by mechanical stress act as signaling molecules, activating plant stress responses. Through signal transduction pathways, they regulate the expression of relevant genes, promoting the synthesis of antioxidants and enhancing the activity of antioxidant enzymes, forming a dynamic feedback regulatory mechanism that enables plants to better adapt to mechanical stress environments. In natural environments, plants often face the combined effects of multiple stress factors simultaneously. Current research on abiotic stress mainly focuses on the impact of drought, high temperature, cold, nutrient deficiency, and excessive salt or toxic metal content in the soil on the plant redox state [41, 53, 54]. The synergistic effects of mechanical stress and co‐occurring abiotic stresses (e.g., drought, salinity) on antioxidant defenses remain underexplored, despite their ecological and agronomic relevance. By integrating multi‐stress experiments with comparative phylogenomics, we can identify conserved and divergent antioxidant strategies that underpin stress adaptation. Such insights will not only advance crop breeding but also illuminate how mechanical pressures have shaped plant evolution over geological timescales.

### 3.2 Antioxidant Defense in Microorganisms: Ecological Optimization of a Minimalist System

Microorganisms constantly face various mechanical stress challenges in nature. For example, deep‐sea archaea live in high‐pressure extreme environments for extended periods [10, 55]. Extreme high pressure disrupts normal cellular metabolic processes, triggering excessive production of ROS. Nevertheless, microorganisms have evolved a delicate and efficient antioxidant defense system to cope with this crisis. Dimethylsulfoniopropionate (DMSP), one of the most abundant organic sulfur molecules on Earth, functions as an antioxidant and high‐pressure protectant, as well as an important carbon and sulfur source for microorganisms. Various deep‐sea bacteria living in high‐pressure environments can synthesize this substance to resist external environmental stimuli [5, 56]. Additionally, some marine bacteria can synthesize vitamin B12 with antioxidant effects [57]. Vitamin B12 participates in various reactions during bacterial metabolism, and its antioxidant effect helps protect bacterial cells from oxidative damage, ensuring normal cellular physiological functions [58]. This strategy differs from that in eukaryotes, which rely on the synergistic action of multiple antioxidants. Instead, bacteria focus on utilizing a single molecule with multiple functions to cope with oxidative challenges, reflecting their uniqueness and innovation during evolution. Pseudomonas aeruginosa lives in environments with fluid shear forces and can enhance its environmental adaptability by degrading H_2_O_2_ through catalase and peroxidase [59]. This strategy of using specific enzymes to scavenge ROS also exists in eukaryotes, such as CAT and APX in plants. From an evolutionary convergence perspective, this indicates that different organisms may independently evolve similar solutions when facing analogous oxidative challenges, namely, degrading ROS through the action of specific enzymes to maintain cellular redox balance. However, bacteria's strategy is relatively simple, mainly relying on the action of a few enzymes, while the antioxidant enzyme systems in eukaryotes are more complex and diverse, involving the synergistic action of multiple enzymes and complex regulatory mechanisms. This reflects that different organisms have developed antioxidant strategies of varying complexity during evolution according to their ecological needs and cellular structural characteristics.

Although the conserved mechanosensors in bacteria, similar to eukaryotic Piezo channels, remain unclear at present, structures such as the cell envelope, flagella, and pili in bacteria play crucial roles in sensing and transmitting mechanical forces [60]. These structures may serve as potential mechanosensors to trigger specific antioxidant responses. The bacterial cell envelope is the interface directly in contact with the external environment. When bacteria experience mechanical stress—such as pressure or shear forces—the resulting deformation of the cell envelope can initiate signal transduction cascades, ultimately eliciting antioxidant defense mechanisms. Studies have shown that changes in the composition and structure of the cell membrane can affect the sensitivity of bacteria to oxidative stress [61, 62], suggesting that the cell envelope may play an important role in mechanosensory sensing and the triggering of antioxidant responses. Flagella are the locomotive organs of bacteria, and their motor structures can generate and sense mechanical forces during bacterial movement [63]. This suggests that mechanical perturbations may be transduced by the flagellar motor into intracellular signals, inducing antioxidant defense responses. Pili are fine protein appendages on the surface of bacteria that play roles in bacterial adhesion and biofilm formation [64]. When bacteria adhere to a surface or interact with other cells through pili, the mechanical forces generated by the pili may activate the intracellular c‐di‐GMP signaling system [60]. The activation of the c‐di‐GMP signaling system can enhance bacterial tolerance to oxidative stress, thereby improving their antioxidant capacity [65]. This suggests that pili may serve as potential mechanosensors to regulate intracellular antioxidant responses.

Although the precise molecular mechanisms by which these potential bacterial mechanosensors trigger specific antioxidant responses are not fully understood at present, an increasing number of studies suggest that they may be of great significance in bacteria's responses to mechanical stress and oxidative stress. With a robust antioxidant defense system, microorganisms can maintain normal physiological functions in harsh mechanical stress environments and occupy a wider range of ecological niches. However, the specific mechanisms by which microorganisms mobilize their antioxidant defense systems in response to mechanical stress, especially the precise mechanisms by which potential mechanosensors trigger antioxidant responses, remain to be further studied. Meanwhile, there are numerous methodological challenges in the field of microbial antioxidant research. First, there are limitations in experimental model construction. Currently, experimental models used to study microbial antioxidant mechanisms are mostly based on laboratory culture conditions, which differ significantly from the complex mechanical stress in natural environments. For example, laboratory‐simulated high‐pressure environments cannot fully replicate the real conditions in the deep sea, which may lead to an inability to accurately reveal the antioxidant response mechanisms of microorganisms under real mechanical stress. Second, the sensitivity and specificity of detection techniques are insufficient. When studying the generation and scavenging mechanisms of ROS during microbial antioxidant processes, existing detection techniques have limitations. ROS have high reactivity and a short half‐life, and commonly used detection methods, such as fluorescence probe methods, are easily affected by multiple factors, limiting the accuracy and reliability of detection results. For the detection of low‐abundance antioxidant‐related molecules and signaling pathways, the sensitivity of existing techniques is also insufficient, restricting an in‐depth understanding of microbial antioxidant mechanisms. Third, the study of microbial communities is complex. In natural environments, microorganisms exist in the form of communities, and their complex interactions can affect the antioxidant capacity and response strategies of individual organisms. However, current research mostly focuses on single microbial species, with relatively few studies on antioxidant mechanisms at the microbial community level, presenting numerous methodological challenges for studying their antioxidant mechanisms.

To more accurately study the antioxidant mechanisms of microorganisms under natural mechanical stress, it is necessary to develop experimental models that are closer to real environments in the future. For example, combining field sampling and laboratory simulation, placing microbial samples collected from natural environments in experimental devices that simulate natural mechanical stress conditions for study. Additionally, microfluidic technology can be used to precisely control the mechanical environmental parameters of microorganisms. To address the shortcomings of detection techniques, it is essential to develop more advanced and accurate detection methods, such as combining nanotechnology and mass spectrometry to develop novel ROS detection probes and platforms, and using high‐throughput sequencing technology and proteomics technology to comprehensively analyze the expression changes of antioxidant‐related genes and proteins in microorganisms under mechanical stress. Given the importance of microbial communities, research on antioxidant mechanisms at the microbial community level should be strengthened. Through metagenomics, metatranscriptomics, and metaproteomics techniques, comprehensively analyze the gene composition, gene expression, and protein function changes of microbial communities under mechanical stress, and reveal the influence mechanisms of interactions among different microorganisms in microbial communities on antioxidant capacity. Moreover, research on the regulation of microbial communities can be carried out to improve the overall antioxidant capacity of microbial communities, providing new strategies for coping with environmental mechanical stress.

### 3.3 Antioxidant Defense in Animals: Multi‐System Integration and Clinical Intervention

Animals have established a sophisticated redox regulatory network to counteract oxidative damage induced by mechanical stress. Mechanical stress is perceived by sensors such as primary cilia [66, 67] or Piezo channels [12, 68], primarily translated into Ca^2^^+^ signals, which subsequently activate antioxidant enzyme systems, including the Nrf2 regulatory axis [69]. This precise regulation is particularly prominent in the cardiovascular system: vascular endothelial cells sense blood flow shear stress through Piezo1 channels, triggering Ca^2^^+^‐dependent signal cascades that activate endothelial nitric oxide synthase (eNOS) to generate nitric oxide (NO) [70, 71]. NO can partially directly bind to superoxide anions and also induce the expression of SOD [72, 73], constructing a dynamic balance system of mechanical perception‐gas signaling‐enzymatic defense.

Although physiological mechanical stress is crucial for the physiological adaptation of animals, abnormal mechanical stress is associated with vascular remodeling in atherosclerosis [7, 74], hypertension [75, 76], vein graft failure [77, 78], and the formation of aortic aneurysm [79]. Excessive ROS induced by mechanical stress can trigger signal transduction events, further exacerbating endothelial dysfunction and vascular remodeling [7, 80]. In thoracic aortic aneurysm (TAA) [81, 82] and abdominal aortic aneurysm (AAA) [81, 82, 83, 84], oxidative stress resulting from ROS production can induce eNOS uncoupling and lead to vascular remodeling. Although enzymatic and non‐enzymatic antioxidant systems also play vital roles in the human body [85, 86], unlike the evolutionary strategies of plants or microorganisms to resist oxidative stress, the ability of animals to cope with extreme mechanical stress is limited by the buffering capacity of their endogenous antioxidant network. For example, clinical interventions such as mechanical ventilation expose the limitations of the animal antioxidant system. We have confirmed that mechanical stress‐induced overexpression of NOX2 promotes oxidative stress in the lungs of ventilated mice, leading to endothelial dysfunction in the mouse lungs, while antioxidant intervention with quercetin can target NOX2 and effectively attenuate ventilator‐induced lung injury [8]. Additionally, N‐acetylcysteine (NAC) maintains glutathione reserves by providing cysteine precursors and scavenges some ROS, inhibiting ventilator‐induced lung injury (VILI) in rats [87]. Animal experiments further support the intervention value of antioxidants: vitamin C can alleviate the oxidative stress state in myocardial ischemia‐reperfusion injury [88]; melatonin enhances mitochondrial antioxidant capacity by activating the SIRT3‐SOD2 pathway [89].

These pieces of evidence suggest that although animals have evolved complex multi‐system antioxidant integration mechanisms, they still require exogenous antioxidant supplementation (compensatory intervention) to fill the gaps in endogenous defense under excessive mechanical stress. However, this contradiction between evolutionary conservatism and clinical intervenability provides a unique perspective for translational medicine: by dissecting the regulatory nodes of the animal antioxidant network (such as Nrf2 ubiquitination modification and Piezo channel gating mechanisms), novel antioxidant therapies can be designed to transform mechanical stress sensitivity into clinical therapeutic targets.

Although animal studies suggest that antioxidants can counteract oxidative damage from mechanical stress, unresolved controversies and methodological limitations in human trials continue to impede their clinical adoption. The timing and dosage of antioxidant therapy are key factors. During early disease progression, oxidative stress may remain below the threshold for substantial cellular damage, and premature antioxidant intervention could interfere with endogenous defense mechanisms. Conversely, in advanced stages, irreversible oxidative injury may limit therapeutic efficacy to symptomatic relief. Furthermore, dosage optimization is critical—insufficient doses fail to neutralize excess ROS, whereas excessive doses risk adverse effects or paradoxical oxidative stress. For instance, high doses of vitamin C may transform into a pro‐oxidant. Moreover, different individuals exhibit variations in metabolism and response to antioxidants [85]. Currently, the absence of large‐scale, long‐term clinical trial data hinders the development of standardized dosage regimens, leaving evidence‐based protocols unestablished. There are also discrepancies between animal models and human clinical scenarios. Although animal models serve as vital research tools, their significant divergence from human pathophysiology underscores the need for complementary clinical studies. The methodologies employed to induce mechanical stress in animal models diverge significantly from human applications. Furthermore, interspecies variations in physiological structure, metabolic pathways, and immune function critically alter the pharmacokinetics (absorption, distribution, metabolism, and excretion) of antioxidants, ultimately impacting their therapeutic efficacy and safety profiles. The long‐term efficacy and safety issues of antioxidant therapy also warrant attention. Current investigations predominantly emphasize short‐term efficacy assessments, yet prolonged antioxidant administration may disrupt physiological homeostasis. Potential mechanisms include interference with cellular signaling pathways, modulation of enzymatic activity, and dysregulation of gene expression. Chronic use could also induce adaptive tolerance, diminishing therapeutic effects. Moreover, systemic accumulation of certain antioxidants—particularly metal‐chelating agents—may elicit organ toxicity.

To address the current controversies and limitations, future research should focus on the following directions: Mechanistic studies, investigate the precise molecular targets and mechanisms of antioxidants to facilitate the development of more specific and effective therapeutic agents. Treatment optimization, conduct large‐scale, long‐term clinical trials to determine optimal dosing, timing, and duration of antioxidant therapy, incorporating personalized medicine approaches. Translational research enhances the relevance of animal models to human clinical settings through improved experimental designs and accelerates the translation of preclinical findings into clinical applications. Long‐term monitoring, establish robust follow‐up systems to assess the sustained efficacy and safety of antioxidant treatments, enabling early detection and management of adverse effects.

## 4 Evolutionary Significance of Antioxidant Strategies

The evolutionary conservation and mechanistic diversity of antioxidant systems have served as critical drivers of biological evolution. These dual characteristics provide an unparalleled framework for investigating fundamental evolutionary questions, including: The emergence of primordial life under oxidative constraints, the co‐evolution of stress response networks and redox homeostasis, the role of oxidative niche adaptation in species diversification, and the molecular basis of ecological specialization. During primordial evolutionary stages, Earth's hostile environment presented formidable physicochemical challenges—intense UV radiation, dramatic thermal oscillations, and persistent mechanical abrasion. These stressors collectively drove rampant ROS generation. At this juncture, the conservatism of antioxidant mechanisms provided crucial clues for exploring the origin of life's stress response systems. Enzymatic antioxidant systems such as SOD and CAT, along with non‐enzymatic antioxidants like ascorbic acid and glutathione, are widely present and highly homologous in eukaryotes [1, 2]. These findings suggest that such antioxidant systems originated during life's primordial evolution and were subsequently conserved as universal protective mechanisms against oxidative stress. For instance, SOD exists in plants, animals, and microorganisms. While SOD isoforms and their subcellular compartmentalization exhibit species‐specific variations, the enzyme's catalytic function—dismutating superoxide radicals into hydrogen peroxide and molecular oxygen—is evolutionarily conserved [6, 31, 32]. This conservatism suggests that during the origin of life, antioxidant mechanisms might have evolved as a basic survival toolkit, ensuring the survival and reproduction of organisms in an environment full of oxidative stress. Moreover, the conservatism of MSCs, such as Piezo channels, in animals and plants also provides strong evidence for the origin of stress response systems. This conserved mechanism of mechanical perception and signal transduction indicates that organisms may have developed a universal strategy during evolution to cope with oxidative stress induced by mechanical stress, namely, by sensing mechanical signals to activate antioxidant defense systems and maintain cellular redox balance.

However, there are significant differences in the tolerance thresholds and signal interpretations of ROS among different species. These context‐specific differences reflect the refined balance of redox regulation during life evolution. The tolerance threshold of plants to ROS is closely related to their ecological adaptation strategies. For example, desert plants enhance their tolerance to osmotic stress induced ROS by accumulating compatible solutes such as proline [90], while aquatic plants rapidly scavenge ROS bursts triggered by external stresses by increasing the activity of antioxidant enzymes such as APX [91]. The ROS tolerance threshold of microorganisms is highly correlated with the extremity of their habitats. DMSP acts as an antioxidant and high‐pressure protectant to maintain ROS concentrations within a physiologically tolerable range [5], while thermophiles ensure efficient ROS scavenging at high temperatures by evolving heat‐stable ROS‐scavenging enzymes such as superoxide dismutase [92]. In animals, under pathological conditions such as mechanical ventilation or atherosclerosis, the burst of ROS in the human body can rapidly exceed the buffering capacity of the endogenous antioxidant network, leading to tissue damage. It is worth noting that some animals, such as naked mole‐rats, have significantly increased their tolerance threshold to ROS by evolving unique antioxidant mechanisms (such as high concentrations of hyaluronic acid), thus extending their lifespan and enhancing their anti‐cancer capabilities [93].

The conservatism and diversity of antioxidant strategies also provide important clues for understanding the origin and evolutionary trajectory of life. From the perspective of the origin of life, the emergence of antioxidant mechanisms may have been a key turning point in life evolution. In the early stages of life evolution, the Earth's environment was harsh, and oxidative stress was one of the major challenges faced by organisms. Organisms that could evolve effective antioxidant mechanisms were more likely to survive in such harsh environments and further evolve into more complex life forms. Therefore, the emergence of antioxidant mechanisms may have provided necessary conditions for the origin and early evolution of life.

From the perspective of evolutionary trajectory, the conservatism and diversity of antioxidant strategies reflect two different strategies of life during evolution: the conservatism strategy ensures the stability and reliability of life in the face of fundamental challenges, while the diversity strategy enables life to adapt to changing environments and ecological niches. These two strategies interact and balance each other, driving the continuous evolution and progress of life. By thoroughly analyzing the conservatism and diversity of antioxidant strategies, we can reveal the mysteries of life evolution from multiple dimensions. This not only deepens our understanding of the essence of life but also provides new directions and ideas for future research in life sciences.

## 5 Conclusions

When facing the ubiquitous challenge of mechanical stress, life exhibits exquisite design: it adopts a conserved chemical (redox) strategy to neutralize ROS and maintain redox balance; however, the implementation of this strategy varies widely across physical and molecular levels. Different species have developed distinct antioxidant strategies based on their ecological requirements and evolutionary histories. From plants utilizing complex internal systems to collaboratively defend against oxidative damage caused by mechanical stress, to microorganisms optimizing antioxidant strategies in minimalist systems to adapt to extreme environments, and further to animals constructing multi‐system integrated antioxidant networks and relying on clinical interventions to compensate for endogenous defense deficiencies, antioxidant systems continuously reshape the interface between living organisms and the mechanical world.

A profound understanding of this ancient evolutionary meta‐strategy holds immense potential in transitioning from fundamental scientific research to practical applications. In the field of crop breeding, based on the modular logic of the antioxidant system, the unique combinations of antioxidant modules that different plants have evolved during long‐term adaptation to mechanical stress provide us with valuable genetic resources. By conducting in‐depth research on the antioxidant mechanisms of wild plants in extreme mechanical environments, we can screen out gene modules with excellent stress‐resistance traits and precisely introduce them into cultivated crops using gene‐editing technology. This is expected to enhance the antioxidant capacity of crops when facing mechanical stresses such as strong winds, heavy rains, and animal grazing, reduce cell damage, maintain normal physiological functions, and thereby improve crop yield and quality. Meanwhile, understanding the signal transduction and regulatory mechanisms among plant antioxidant modules allows us to optimize the response speed and intensity of the crop antioxidant system by regulating relevant signaling pathways, enabling crops to respond more rapidly and effectively to mechanical stress and enhancing their stress resistance and adaptability.

In the aspect of biomaterial design, the conservatism of antioxidant strategies has inspired the design of intelligent self‐healing biomaterials. The minimalist and efficient antioxidant defense systems that microorganisms have evolved under extreme mechanical stress environments, such as the synthesis of DMSP by deep‐sea archaea as an antioxidant and high‐pressure protectant, can inspire us to introduce molecules or compounds with similar functions into biomaterials. This would enable the materials to automatically generate antioxidant substances when facing mechanical stress, neutralize ROS, and prevent material failure caused by oxidative damage. The dynamic equilibrium system of mechanosensing‐gas signaling‐enzymatic defense in the animal cardiovascular system also provides ideas for designing materials with similar signal‐sensing and response mechanisms to achieve self‐repair and performance recovery. Furthermore, by comparing and analyzing the antioxidant mechanisms of plants, microorganisms, and animals, and integrating the advantages and characteristics of cross‐species antioxidant strategies, we are able to design novel biomaterials with superior performance and greater adaptability. For instance, integrating plants' complex synergistic defense networks with microorganisms' minimalist antioxidant strategies could yield biomaterials with multi‐level antioxidant protection. Alternatively, mimicking animals' multi‐system integrated networks may enable adaptive antioxidant responses under varying mechanical stresses, enhancing material reliability in complex environments.

In the agricultural field, it can provide a solid theoretical foundation for cultivating crops with high mechanical resilience, enabling crops to better withstand the onslaught of harsh environments and ensuring global food security. In the field of materials science, it can offer a continuous stream of inspiration for the design of intelligent self‐healing biomaterials, promoting the research, development, and application of new materials to meet the growing demand for high‐performance materials. In the medical field, it also lays the foundation for the development of new antioxidant therapies, which are expected to be used in the treatment of various diseases caused by mechanical stress, such as ventilator‐induced lung injury and myocardial ischemia‐reperfusion injury, bringing new hope for human health.

In summary, in‐depth research on antioxidant strategies presents a broad prospect, ranging from revealing fundamental molecular mechanisms to addressing major ecological and applied challenges, and will make significant contributions to sustainable human development and the advancement of life sciences.

## Funding

This research was supported by grants from the National Natural Science Foundation of China (Grant No. 12302409) Shanghai Jiao Tong University Medical‐Engineering Interdisciplinary Research Fund (YG2025QNB36).

## Conflicts of Interest

The authors declare no conflicts of interest.

## Data Availability

The authors have nothing to report.
